# Lack of cyclin D3 induces skeletal muscle fiber-type shifting, increased endurance performance and hypermetabolism

**DOI:** 10.1038/s41598-018-31090-5

**Published:** 2018-08-24

**Authors:** Silvia Giannattasio, Giacomo Giacovazzo, Agnese Bonato, Carla Caruso, Siro Luvisetto, Roberto Coccurello, Maurizia Caruso

**Affiliations:** 1 National Research Council (CNR), Institute of Cell Biology and Neurobiology, IRCCS Santa Lucia Foundation, Rome, 00143 Italy; 2 University of Tuscia, Department of Ecological and Biological Sciences, Viterbo, 01100 Italy

## Abstract

The mitogen-induced D-type cyclins (D1, D2 and D3) are regulatory subunits of the cyclin-dependent kinases CDK4 and CDK6 that drive progression through the G1 phase of the cell cycle. In skeletal muscle, cyclin D3 plays a unique function in controlling the proliferation/differentiation balance of myogenic progenitor cells. Here, we show that cyclin D3 also performs a novel function, regulating muscle fiber type-specific gene expression. Mice lacking cyclin D3 display an increased number of myofibers with higher oxidative capacity in fast-twitch muscle groups, primarily composed of myofibers that utilize glycolytic metabolism. The remodeling of myofibers toward a slower, more oxidative phenotype is accompanied by enhanced running endurance and increased energy expenditure and fatty acid oxidation. In addition, gene expression profiling of cyclin D3−/− muscle reveals the upregulation of genes encoding proteins involved in the regulation of contractile function and metabolic markers specifically expressed in slow-twitch and fast-oxidative myofibers, many of which are targets of MEF2 and/or NFAT transcription factors. Furthermore, cyclin D3 can repress the calcineurin- or MEF2-dependent activation of a slow fiber-specific promoter in cultured muscle cells. These data suggest that cyclin D3 regulates muscle fiber type phenotype, and consequently whole body metabolism, by antagonizing the activity of MEF2 and/or NFAT.

## Introduction

D-type cyclins (cyclin D1, D2 and D3) are induced by extracellular stimuli, and are therefore regarded as sensors that link the extracellular environment to the core cell cycle machinery. Once induced, D-type cyclins associate with the partner kinases CDK4 and CDK6 and initiate phosphorylation of the retinoblastoma protein (pRb), which binds to and regulates E2F transcription factors during the early G1 phase of the cell cycle. The subsequent induction of cyclin E and its association with CDK2 is responsible for extensive pRb phosphorylation, release of E2F factors, and thus transcription of genes that are required for the G1/S-phase transition in the cell cycle^1–3^.

Besides pRb, cyclin D-CDK4/6 complexes target other proteins for phosphorylation, including several transcription factors, thus linking the cell cycle and transcription^4–6^. Furthermore, there is increasing evidence that D-type cyclins can play transcription regulatory functions that do not involve the associated kinase activity. This suggests that these cyclins may be involved in different cellular processes in addition to their well-established cell cycle role^6–18^.

Indeed, D-cyclins can be activated by stress, nutrients and hormones, even in non-proliferating cells, and can function as key regulators of cell metabolism^19^. For example, cyclin D1 has been shown to inhibit mitochondrial function, hepatic fatty acid oxidation and lipogenesis, gluconeogenesis and adipogenesis^13,20–24^. Cyclin D2 critically regulates islet β-cell postnatal growth and glucose homeostasis^25,26^. Cyclin D3 promotes adipogenesis and pancreatic β-cell fitness and viability^27,28^.

D-cyclins are highly homologous suggesting redundancy in their functions. However, they also perform unique functions, as indicated by different tissue-specific expression patterns, different induction by various signals in a cell lineage-specific manner, different substrate specificity, and different phenotypic consequences of cyclin D knockouts in mice^5,29–33^.

Earlier studies conducted by us and others in the C2 myogenic cell line suggested that, among the three D-type cyclins, cyclin D3 may play extended functions outside cell-cycle regulation during the process of myogenic differentiation. In fact, in contrast to cyclins D1 and D2, the expression of cyclin D3 is induced in differentiating myocytes at the transcriptional and post-transcriptional level by MyoD and pRb-mediated mechanisms, respectively^34–39^.

In skeletal muscle, cyclin D3 is expressed at high levels during the late stages of fetal development and early postnatal life but not in fully developed myofibers, suggesting a role for cyclin D3 in the establishment rather than maintenance of terminal myogenic differentiation^40^. We have recently shown that adult skeletal muscle of cyclin D3-knockout mice displays a reduction in myofiber number and size and in the number of skeletal muscle-specific stem cells (satellite cells), suggesting that cyclin D3 is involved in the satellite cell-mediated muscle growth and in the establishment of a proper pool size of adult satellite cells occurring during post-natal development^41^. Furthermore, the analysis of satellite cells *in vitro* and their response to *in vivo* muscle injury revealed that the absence of cyclin D3 results in reduced proliferation and precocious differentiation of myogenic progenitors, indicating that cyclin D3 critically controls the balance between myoblast proliferation, differentiation and self-renewal^41^. Given that proliferation and differentiation of muscle precursor cells are mutually exclusive processes, cyclin D3 might play a crucial role in the modulation of cell cycle and/or in regulating the expression of muscle differentiation genes.

Skeletal muscle is the largest tissue of the body playing a central role not only in motility but also in the control of whole-body metabolism^42^. The body’s musculature is composed of a variety of muscle groups, each containing heterogeneous myofibers with distinct biochemical, contractile and metabolic properties, thus enabling different muscle groups to perform specific motor activities. Myofibers are classified into slow-twitch, type I, and fast-twitch, type IIa, type IIx and type IIb fibers, based on contractile performance and on the expression of specific isoforms of myosin heavy chain. Slow/type I and fast/type IIa myofibers exhibit oxidative metabolism, are resistant to fatigue and are highly vascularized to ensure a steady supply of oxygen and nutrients. Fast/type IIx and IIb myofibers display progressively lower oxidative capacity, utilize predominantly glycolytic metabolism, are quickly fatigable and have a reduced blood supply^43^. Muscle fiber type diversification is established during embryonic development and reflects different patterns of gene expression, but the adult fiber type profile emerges during early postnatal life as a result of the maturation of neuromuscular junctions and under the influence of thyroid hormone^43^. In adult muscle, specialized myofibers remain plastic and can adapt their metabolic and contractile profile in response to stressful conditions and functional demands, such as exercise, motor neuron activity and metabolic challenges^44–46^.

Given the emerging role of D-type cyclins in the control of metabolic processes and transcription programs, the present study was undertaken to assess whether lack of cyclin D3 affects whole body energy homeostasis as well as the gene expression, the metabolic profile and the motor performance of skeletal muscle.

## Results

### Cyclin D3 inactivation results in enhanced energy expenditure

**Figure 1:**
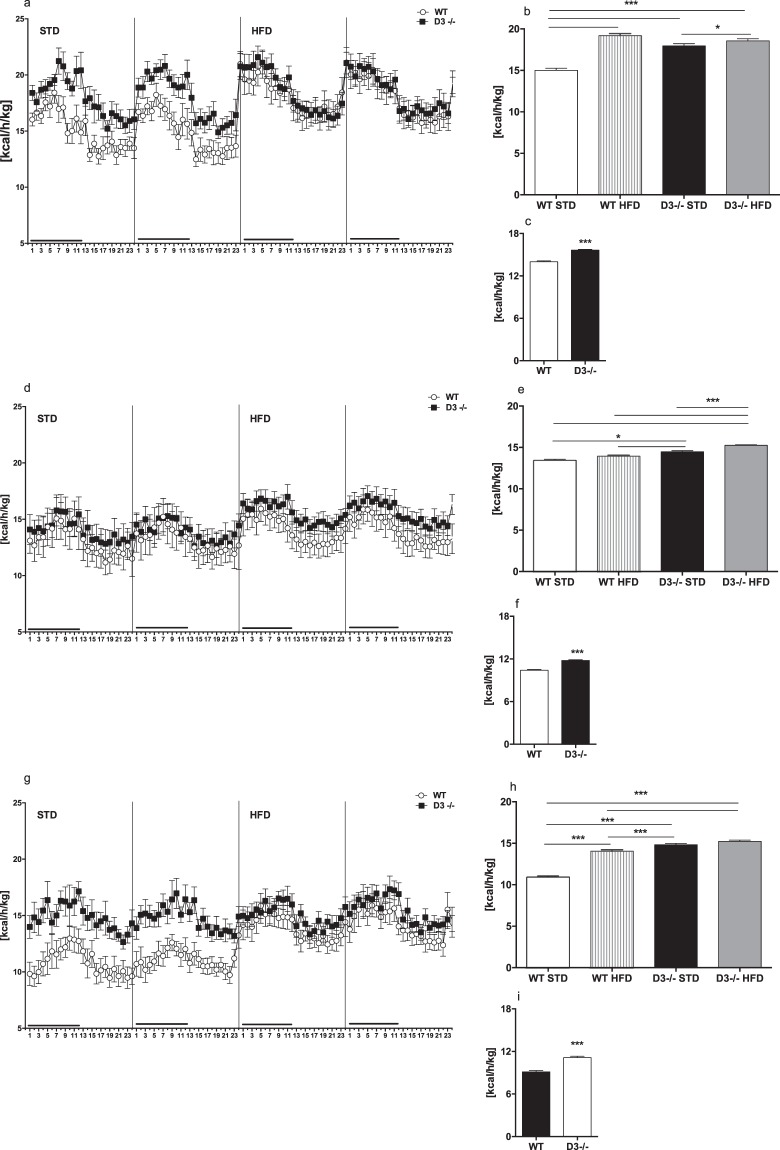
Energy expenditure. Upper (**a**–**c**), middle (**d**–**f**), and lower (**g**–**i**) panels are referring to juvenile (D3−/−, n = 12; WT, n = 10), adult (D3−/−, n = 11; WT, n = 7) and aged (D3−/−, n = 8; WT, n = 9) mice, respectively. (**a**,**d**,**g**) Four days of continuous indirect calorimetry recording. Mean EE is reported in terms of heat (kcal/kg) emitted for each hour per day (24 time bins of 1 h each) in STD (two days) or HFD (two days) feeding conditions. The horizontal black bar at the beginning of each 24hrs cycle indicates the dark cycle period. (**b**,**e**,**h**) Mean of two days of EE produced by WT and D3−/− mice in STD or HFD feeding conditions. Two-way ANOVA analysis of EE data showed significant main effects for groups and time in juvenile (groups: F_3,1730_ = 135.9, p < 0.0001; time: F_46,1730_ = 10.17, p < 0.0001), adult (groups: F_3,2201_ = 31.61, p < 0.0001; time: F_70,2201_ = 1.79, p < 0.0001) and aged mice (groups: F_3,1410_ = 183.9, p < 0.0001; time: F_46,1410_ = 3.67, p < 0.0001). The analysis showed also significant groups x time interactions in juvenile, adult and aged mice (F_138,1730_ = 1.40, p < 0.001; F_210,2201_ = 1.19, p < 0.001; F_138,1410_ = 1.35, p < 0.001, respectively). Post hoc test for multiple comparisons data were performed by Bonferroni’s test. The bars above histograms depict the comparisons between genotypes and feeding conditions. Asterisks denote significance (*p < 0.01; ***p < 0.0001). (**c**,**f**,**i**) Mean of four days of resting EE (REE) produced by WT and D3−/− mice in both STD and HFD feeding conditions. Two-tailed unpaired *t*-tests showed significant increase of REE in D3−/− mice irrespective of motor activity (***p < 0.0001 vs WT). The error bars in histograms represent standard errors of the means (SEM).

To investigate the role of cyclin D3 in the control of energy homeostasis, we assessed the metabolic rate (energy expenditure, EE) of cyclin D3−/− (hereafter referred to as D3−/−) and WT male mice by means of indirect calorimetry during 4 days of continuous analysis/recording. The analysis was carried out in animals at three different ages: 2–3 (juvenile), 8–10 (adult), and 22–24 (aged) months, respectively. Mice were analyzed during the first 2 days in standard nutritional conditions (standard diet, STD), and then shifted to a high fat diet (HFD) regimen during the remaining two days (Fig. 1, panels a,d,g). Two-way ANOVA analysis of EE data from juvenile, adult and aged mice showed significant main effects for groups and time (Fig. 1 legend).

As revealed by post-hoc analysis, D3−/− mice showed enhanced EE (i.e., increased heat production) in STD conditions as compared to their WT counterparts, which appeared less marked (although significant) in adult mice (Fig. 1, panels b,e,h).

Exposure to high-calorie diet (i.e., HFD) increased EE in juvenile and aged WT animals (Fig. 1, panels b,h, respectively), while no effect of HFD was observed in adult WT animals (Fig. 1, panel e). On the other hand, after exposure to HFD, D3−/− mice exhibited a further increase of EE at juvenile and adult ages but not at old age (Fig. 1, panels b and e vs. panel h). Of note, the levels of EE in HFD-fed D3−/− mice were either higher (adult and aged mice; Fig. 1, panels e and h, respectively) or equivalent (juvenile mice; Fig. 1, panel b) to those displayed by control WT mice under the same high caloric diet regimen.

The analysis of EE in resting conditions (REE), thus considering only EE values generated in lack of motor activity (i.e., 0–3 counts), showed the constant increase of heat production in D3−/− mice at all ages examined (Fig. 1, panels c, f and i).

As for the total amount of diet consumed, the intake of D3−/− mice was lower than in WT mice for both diets. However, to further examine this aspect, we calculated and compared the food efficiency ratio (FER) in both genotypes in HFD-fed mice. FER was computed as body weight change (g)/food intake (g) during the 48-h of HFD exposure. FER was not different between the two genotypes in young (0.636 ± 0.012 (WT) and 0.529 ± 0.145 (D3−/−)), adult (0.699 ± 0.223 (WT) and 0.824 ± 0.134 (D3−/−)) and aged mice (0.625 ± 0.131 (WT) and 0.708 ± 0.097 (D3−/−)).

**Figure 2:**
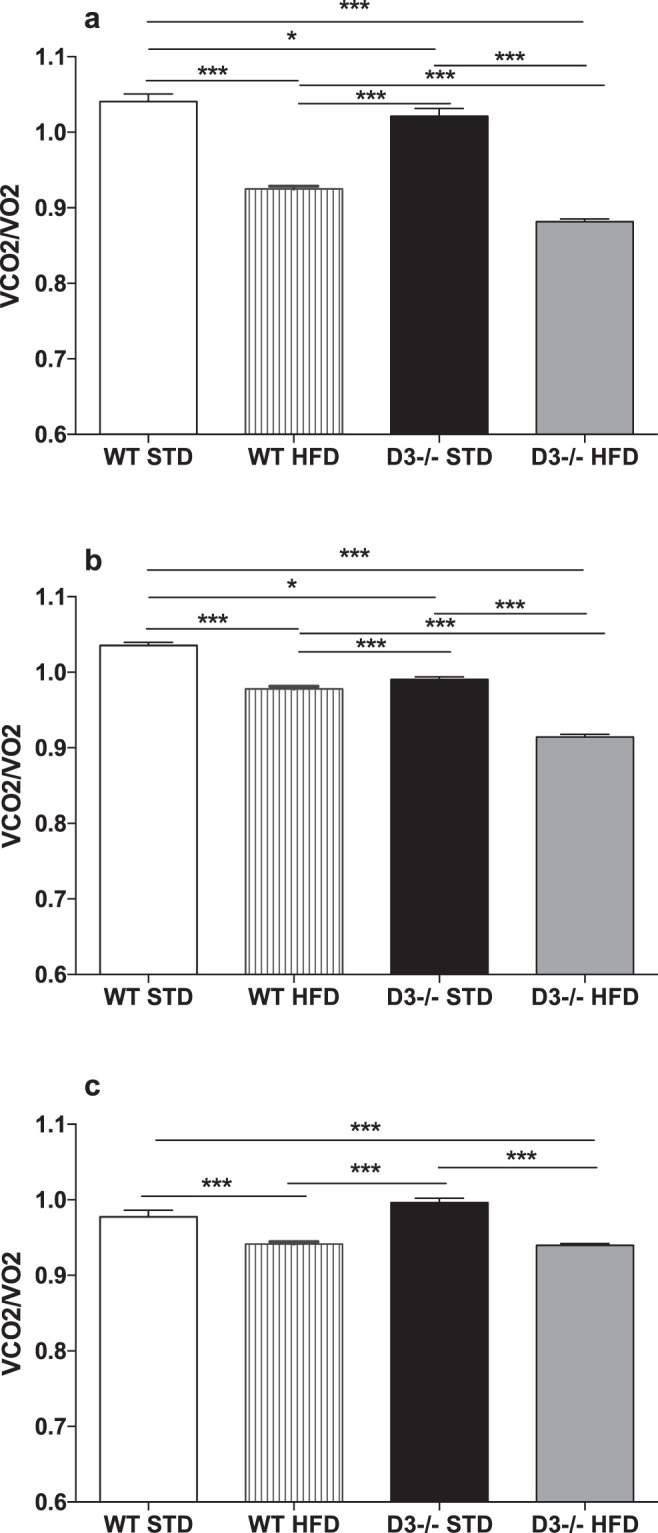
Respiratory exchange ratio. Upper (**a**), middle (**b**), and lower (**c**) panels are referring to juvenile (D3−/−, n = 12; WT, n = 10), adult (D3−/−, n = 11; WT, n = 7) and aged (D3−/−, n = 8; WT, n = 9) mice, respectively. RER (*V*CO_2_/*V*O_2_) as the extent of lipid vs. carbohydrate oxidation in STD or HFD feeding conditions in D3−/− and WT mice. Two-way ANOVA analysis of RER data showed significant main effects for groups and time in juvenile (groups: F_3,1880_ = 544.2, p < 0.0001; time: F_46,1880_ = 14.76, p < 0.0001), adult (groups: F_3,1440_ = 143.1, p < 0.0001; time: F_47,1440_ = 1.57, p < 0.01) and aged mice (groups: F_3,1410_ = 41.31, p < 0.0001; time: F_46,1410_ = 3.54, p < 0.0001). The analysis showed also significant groups x time interactions (F_138,1880_ = 2.11, p < 0.0001; F_141,1440_ = 1.37, p < 0.01; F_138,1410_ = 2.04, p < 0.0001 as for juvenile, adult and aged mice, respectively). Post hoc tests for multiple comparisons data were performed by Bonferroni’s test. The bars above histograms depict the comparisons between genotypes and feeding conditions. Asterisks denote significance (*p < 0.01; ***p < 0.0001). The error bars in histograms represent standard errors of the means (SEM).

Next, we collected data on the respiratory exchange ratio (RER) to evaluate which fuel among carbohydrates, fat or proteins is predominantly oxidized (Fig. 2). RER stands for ratio of volume of CO2 produced to the volume of O2 used (VCO2/O2), and increases when carbohydrates are primarily used while decreases in case more O2 should be consumed to achieve lipid oxidation.

The full two-way ANOVA analysis of RER for juvenile, adult and aged mice is reported in Fig. 2 legend. As confirmed by post-hoc analysis, the exposure to HFD reduced RER values irrespective of the genotype or age analyzed (Fig. 2, panels a, b, c). Moreover, in D3−/− HFD-fed juvenile and adult mice, the reduction was more pronounced than in WT HFD-fed mice (Fig. 2, panels a and b), thus indicating in D3−/− mice a higher than in WT mice reactivity to fatty diets challenge. Remarkably, juvenile and adult D3−/− mice exhibited lower RER values than WT mice also in standard feeding conditions (WT STD vs. D3−/− STD; Fig. 2, panels a,b), which is indicative of an enhanced level of fatty acid oxidation and prevalent use of fatty acids as fuel source even when feeding does not involve a hyperlipidic diet.

As for D3−/− HFD-fed aged mice, RER values were lower than in STD-fed mice but not in comparison to WT HFD-fed mice (Fig. 2, panel c). The difference between juvenile and adult HFD-fed D3−/− mice and aged HFD-fed D3−/− mice might be due to a general decline of the adaptive metabolic response to the HFD challenge as a function of aging (Fig. 2, panels a and b vs. panel c; RER mean D3−/− HFD juvenile and adult 0.87 and 0.91, respectively, vs. RER mean D3−/− HFD aged 0.94). On the other hand, RER values did not differ between aged D3−/− and WT groups also under STD feeding conditions, which might indicate an age-related decline in the ability of D3−/− mice to maintain the higher level of lipid oxidation displayed at younger ages.

Altogether, the above data indicate that in standard nutritional conditions both EE and REE levels were enhanced in D3−/− mice irrespective of age, while fat oxidation capacity was enhanced in juvenile and adult D3−/−mice and appeared to decline with age.

### Cyclin D3 inactivation results in improved endurance exercise performance

Skeletal muscle accounts for approximately 40% of total body weight and represents the major source of total EE in mammals. Since the proportion of glycolytic and oxidative myofibers plays a pivotal role in overall metabolism, we reasoned that the loss of cyclin D3 might promote a more oxidative muscle fiber type phenotype.

**Figure 3:**
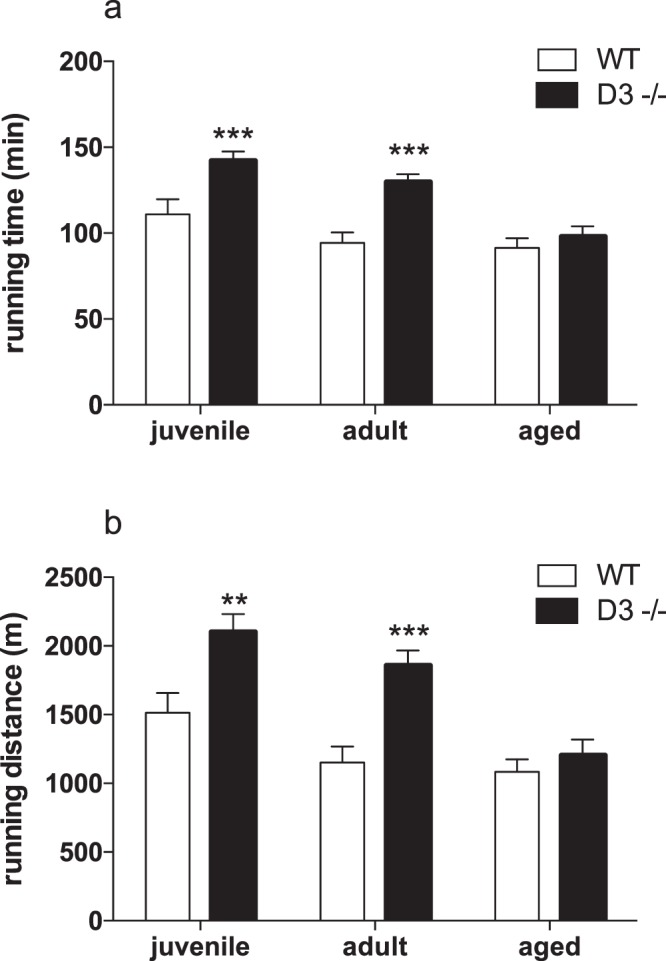
Lack of cyclin D3 improves exercise endurance. Running time (panel a) and running distance (panel b) were acquired in juvenile (D3−/−, n = 13; WT, n = 12), adult (D3−/−, n = 14; WT, n = 12), and aged mice (D3−/−, n = 11; WT, n = 10) during a treadmill endurance exercise as described in methods. Data are reported as means+/− SEM and statistical difference between WT and D3−/− mice were calculated by two-way ANCOVA, using body weight as a covariate, followed by Fisher PLSD *post-hoc* comparison of age matched D3−/− vs. WT mice. Compared to WT mice, both running time and running distance were significantly increased in D3−/− juvenile (**p < 0.01) and D3−/− adult (***p < 0.001), but not in D3−/− aged mice.

A functional hallmark of slow and fast/oxidative myofibers is their fatigue resistance and capacity to sustain prolonged duration of low-intensity muscle activity. Thus, we examined the exercise capacity of juvenile, adult or aged D3−/− male mice during an endurance-type running challenge, as compared to age- and sex-matched WT littermates (Fig. 3). Mice were forced to run on a up-hill treadmill until exhaustion; the time to exhaustion closely correlates with oxidative capacity.

The endurance exercise revealed that juvenile and adult D3−/− mice could run for a longer time than age-matched WT mice (Fig. 3, panel a). Moreover, juvenile and adult D3−/− mice demonstrated the ability to travel longer distances than age-matched WT mice (Fig. 3, panel b). The improved physical endurance of D3−/− mice was not observed in aged mice, which did not perform differently from age-matched WT mice (Fig. 3, panels a,b).

Although D3−/− mice were smaller in body weight compared to age-matched WT mice (18.46 ± 0.57 vs. 25.33 ± 0.59; 25.61 ± 1.09 vs. 30.31 ± 1.26 and 25.27 ± 0.93 vs. 31.80 ± 0.90 g, for juvenile, adult and aged mice, respectively), the genotype effect on running time and running distance remained statistically significant after inclusion of body weight as covariate in a two-way ANCOVA analysis (distance: F_1,60_ = 5.523, p = 0.0221; time: F_1,60_ = 4.417, p = 0.0398).

Both for running time and running distance, post hoc comparisons between D3−/− and WT littermates confirmed significant differences in juvenile (time: p = 0.0009; distance: p = 0.0021) and in adult (time: p = 0.0002; distance: p = 0.0002), but not in aged mice.

Overall, our data indicate that the absence of cyclin D3 improves performance during a single bout of treadmill endurance-type exercise, which suggest a higher oxidative capacity of skeletal muscle.

Nevertheless, the enhanced fatigue-resistance exhibited by D3−/− mice is lost with the increasing age of mice. Interestingly, D3−/− mice also exhibit an age-dependent decline in their preference for lipids usage as fuel source relative to WT mice (Fig. 2, panel c), thus suggesting a possible link between decreased ability to utilize high-energy fuels (i.e., lipids) and decreased endurance performance.

### Cyclin D3 regulates the myofiber phenotype

**Figure 4:**
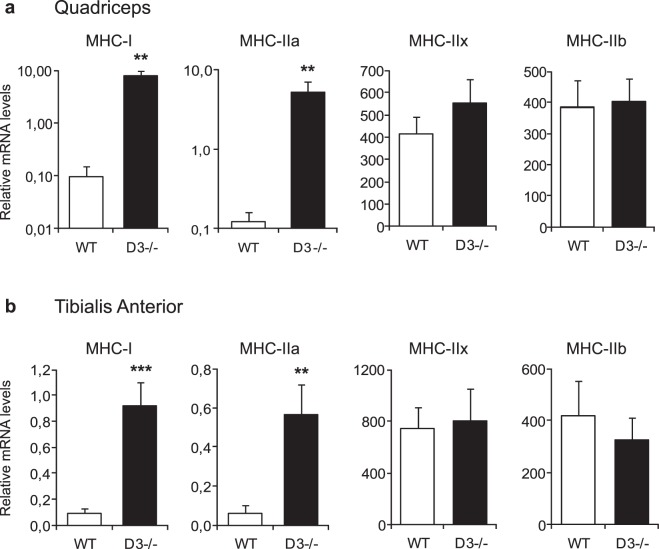
Cyclin D3 regulates the expression of slow, oxidative MHC isoforms. Transcripts coding for MHC-I (*Myh7*), MHC-IIa (*Myh2*), MHC-IIx (*Myh1*) and MHC-IIb (*Myh4*) were determined in quadriceps (panel a) and tibialis anterior (panel b) muscles of adult D3−/− and WT mice by quantitative real-time PCR (n = 7 per group). mRNA levels are expressed relative to TBP (TATA binding protein) mRNA, used as endogenous control. Data are reported as means ± SEM. Statistical differences between WT and D3−/− mice was assessed by two-tailed unpaired t-tests (**p < 0.01, ***p < 0.001).

To investigate a potential involvement of cyclin D3 in regulating muscle fiber type composition, we analyzed the expression profile of myofiber type-specific isoforms of myosin heavy chain (MHC) in the fast-twitch quadriceps and tibialis anterior (TA) muscles of adult D3−/− mice compared with WT littermates (Fig. 4, panels a,b, respectively). Specifically, we quantified the transcripts encoding the MHC type I isoform, typically expressed in slow-twitch myofibers, and the MCH type IIa, type IIx and type IIb isoforms, typically expressed in fast-twitch myofibers, exhibiting oxidative, intermediate (oxidative/glycolytic) or glycolytic metabolism, respectively. The results indicated an increase in the relative expression of transcripts encoding slow MHC-I and fast/oxidative MHC-IIa myosin heavy chain isoforms in cyclin D3−/− muscles compared with WT muscles, whereas no significant change was observed in the levels of transcripts encoding the fast type IIx and type IIb MHC isoforms.

**Figure 5:**
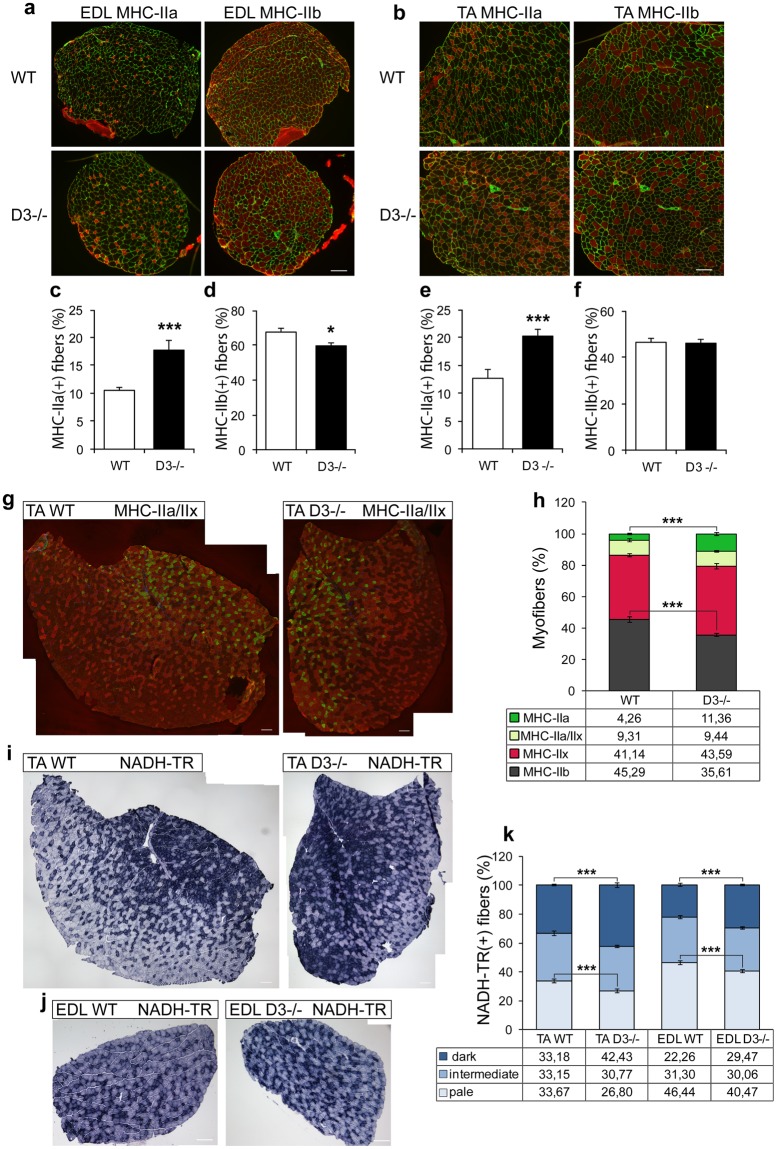
Cyclin D3 regulates muscle fiber type. (**a**,**b**) Immunoistochemical analysis of MHC isoforms in EDL and TA muscles of adult D3−/− and WT mice. Tranverse serial sections of EDL (panel a) or TA muscles (panel b) co-immunostained for laminin (green) to visualize myofiber profiles and either MHC-IIa or MHC-IIb (red). Shown are entire EDL sections and fields of the TA red region. (**c**–**f**) Quantification of MHC-IIa and MHC-IIb fibers in EDL (panels c,d) and TA (panels e,f) serial sections (EDL: WT, n = 4; D3−/−, n = 4; TA: WT, n = 4; D3−/−, n = 5). (**g**) Transverse sections of WT and D3−/− TA muscles co-immunostained for MHC-IIa (green) and MHC-IIx (red) and couterstained with DAPI. (**h**) Quantification of MHC-IIa, MHC-IIx, MHC-IIa/IIx and MHC-IIb (unstained) fibers in WT and D3−/− TA sections (WT, n = 4; D3−/−, n = 4). (**i**, **j**) Histochemical staining of NADH-TR enzymatic activity in transverse sections of WT and D3−/− TA (panel i) and EDL (panel j) muscles. (**k**) Quantification of the number of differentially stained NADH-TR-positive fibers in WT and D3−/− TA and EDL muscle sections (WT, n = 4; D3−/−, n = 4). The data in graphs shown in panels (c–f,h,k), are expressed as the percentage in each category of the total number of fibers per section and are presented as means ± SEM. Quantification was carried out in least three sections per individual mouse. Statistical differences between WT and D3−/− mice were assessed by two-tailed unpaired t-tests (***p < 0.001, *p < 0.05). Scale bar: 100 μm.

The enhanced expression of genes specific to slow and fast/oxidative myofibers may indicate an increase in the number of these myofiber types in cyclin D3−/−muscle. To address this issue, we examined, by immunostaining with monoclonal antibodies specific to the different MHC isoforms, the extensor digitorum longus (EDL) and TA muscles, containing primarily fast type IIx and IIb myofibers (Fig. 5), as well as the soleus, a typical slow muscle composed primarily of slow-type I and fast/oxidative type IIa myofibers (Supplementary Figure S1).

Immunostaining of EDL and TA cross-sections against MHC-IIa revealed a significant increase in the percentage of fast/oxidative myofibers in D3−/− EDL (p = 0.00011) and TA muscles (p = 0.0007) (Fig. 5, panels c,e, respectively). Immunostaining against MHC-IIb, the fastest isoform of MHC, showed a reciprocal decrease in the percentage of fast/glycolytic myofibers in D3−/− EDL muscle (p = 0.0146) (Fig. 5, panel d). By contrast, no significant change was observed in the abundance of type IIb fibers in TA muscle (Fig. 5, panel f).

We reasoned this might be due to the presence of intermediate, hybrid fibers expressing next-neighboring MHC isoforms (i.e. IIb and IIx or IIx and IIa) that normally represent a low percentage of total fibers in a muscle, but can become particularly abundant when a fiber type shift takes place. Therefore, TA muscle sections were co-immunostained with anti-MHCIIa and anti-MHC-IIx monoclonal antibodies, which are of different isotype (Fig. 5, panel g). This staining procedure enabled identification of fibers expressing type IIa (green), type IIx (red) or both type IIa/IIx MHC, whereas the unstained fibers essentially represented fibers expressing exclusively type IIb MHC, as the number of slow MHC-I fibers is extremely low in TA muscle (below the 1% of total fibers, data not shown).

The results not only confirmed a significant increase of MHC-IIa fibers in D3−/− TA muscle (p = 0,000001), but also showed a concomitant decrease of fibers expressing only MHC-IIb (unstained fibers) (p = 0,000095), without an apparent change in the number of fibers expressing MHC-IIx or both MHC-IIa and IIx (Fig. 5, panel h). Moreover, the results also revealed the presence of nearly 10% of hybrid MHC-IIb/IIx fibers in D3−/− muscle, as calculated by subtracting the number of unstained fibers from the number of MHC-IIb-positive fibers detected by immunostaining against MHC-IIb (Fig. 5, panel b), with a consequent decrease of fibers expressing only MHC-IIx.

Altogether, the above data clearly indicate a progressive fast-to-slow fiber type switch in the direction IIb⤍IIx⤍IIa in both the EDL and TA fast-twitch muscles of D3−/− mice compared with WT mice.

To ascertain that the observed fast-to-slow shift in fiber type composition corresponds to an increase of myofibers utilizing oxidative metabolism, EDL and TA muscles were further assessed by histochemical staining for the oxidative enzyme NADH tetrazolium (NADH-TR), which is in general high in MHC-IIa fibers, intermediate in type IIx fibers and low in type IIb fibers. The number of NADH-TR-positive fibers and the staining intensities were increased in D3−/− TA and EDL muscles (Fig. 5, panels i, j, k), indicating a fiber-type transition toward a more oxidative phenotype.

Concerning the soleus muscle, which is already fully oxidative, immunostaining cross-sections with anti-MHCI or anti-MHC-IIa showed a modest, though significant, increase of type I slow myofibers in D3−/− mice relative to their WT counterparts (p = 0.0430), whereas no significant change was observed in the relative abundance of type IIa myofibers (Supplementary Figure S1, panels a, b). We also measured the size of type I and type IIa fibers, which revealed a reduction in the area of both fiber types in D3−/− compared with WT soleus. Interestingly, however, type IIa fibers resulted smaller than type I fibers in D3−/− soleus, whereas the size of the two fiber types did not differ in WT soleus, which indicated a greater ratio of type I to type II fiber mass in D3−/− relative to WT soleus muscles (Supplementary Figure S1, panel c), and thus a fast-to-slow shift in fiber size.

### Ablation of cyclin D3 leads to extensive changes of the muscle transcriptome

To shed light into the molecular pathways deranged in the absence of cyclin D3 and to identify key target genes modulating these pathways, we performed RNA sequencing on quadriceps muscles isolated from D3−/− and WT adult mice (n = 3).

The differential gene expression analysis of RNA-seq results identified a total of 655 genes showing significantly deregulated expression in muscles of D3−/− mice compared with WT mice (q-value < 0.05), of which 530 genes were induced (Supplementary Table S1). Gene ontology (GO) analysis of upregulated transcripts (fold induction ≥1.45) was used to identify terms enriched in “biological processes”, “cellular component” or “molecular function” annotations. Table 1 provides a selected subset of the most relevant GO terms enriched in D3−/− muscle. The entire list can be found in Supplementary Table S1. Considering that cyclin D3 is widely expressed and that skeletal muscle is a complex organ composed of a variety of cell types in addition to myofibers, including blood and endothelial cells, fibroblasts in the connective tissue and nerves, it was not surprising to find out that germline mutation of cyclin D3 influenced the expression of genes participating in diverse biological processes. In fact, in addition to terms related to “striated muscle tissue development and function”, GO analysis of transcripts upregulated in cyclin D3−/− muscles also highlighted the enrichment of gene sets involved in “immune response”, “vasculature development” and various metabolic and signaling pathways. In particular, the highly enriched GO term “immune response” comprised genes involved in innate and adaptive immunity, complement activation and inflammation. It is likely that altered expression of these genes occurs in circulating or muscle resident immune cells because cyclin D3 has been shown to play a crucial role for proper development of several hematopoietic lineages, including immature T lymphocytes, early B cells, germinal center B cells, granulocytes and terminally differentiating erythroid precursors^32,47–51^. Furthermore, the GO analysis for Cellular Component and Molecular Function revealed the marked enrichment of sets of genes encoding proteins of the extracellular matrix and sarcomeric and actin-associated cytoskeletal proteins involved in myofibril assembly and regulation of contractile function. Focusing our attention on GO terms related to striated muscle development/differentiation, muscle function and metabolism, we assembled a representative sample of genes that are activated by cyclin D3 depletion (shown in Supplementary Table S2).

**Table 1:** Gene Ontology analysis of genes upregulated in cyclin D3−/− muscle.

Biological Process	N. of genes	P-Value
immune response	40	1,22E-12
cell adhesion	36	3,60E-08
blood vessel development	18	3,39E-05
muscle organ development	15	4,02E-05
response to oxygen levels	9	9,33E-05
regulation of phosphate metabolic process	19	1,46E-04
striated muscle cell differentiation	9	8,23E-04
positive regulation of cell proliferation	16	1,80E-03
regulation of muscle contraction	5	1,28E-02
glucose metabolic process	8	3,73E-02
regulation of lipid metabolic process	5	4,01E-02
**Cellular Component**	**N. of genes**	**P-Value**
extracellular matrix	36	8,94E-14
myofibril	12	1,87E-05
sarcomere	10	1,89E-04
actin cytoskeleton	16	2,37E-04
I band	7	2,16E-03
**Molecular Function**	**N. of genes**	**P-Value**
calcium ion binding	46	6,01E-08
actin binding	21	9,05E-06
insulin-like growth factor binding	5	1,38E-03

Gene Ontology analysis for biological processes, cellular component and molecular function of genes upregulated by ≥1,45-fold in muscles of D3−/− mice versus WT mice. Terms with at least 5 genes associated genome-wide and an enrichment P-Value < 0,05 were evaluated. Additional information in Supplementary Table S1.

Notably, a recent gene expression analysis at the single fiber level allowed the definition of gene expression signatures in slow/oxidative and fast/glycolytic fiber types^52^. By comparing the list of genes upregulated in cyclin D3−/− muscle to the list of genes preferentially expressed in slow myofibers, we observed a considerable number of overlapping genes (Table 2). These genes include many of those already identified through the above GO analyses, namely: (*i*) the slow isoforms of sarcomeric components; (*ii)* slow fiber-specific sarcoplasmic reticulum Ca^2+^ channels and Ca^2+^ binding proteins involved in excitation-contraction coupling; (*iii)* nuclear proteins containing PDZ, LIM, or ankyrin domains that are also found in the sarcomere and (*iv)* transcription regulators involved in fatty acid metabolism. Furthermore, additional genes categorized as slow myofiber-specific by Chemello *et al*.^52^, were found upregulated in cyclin D3−/− muscle.

**Table 2:** Slow myofiber-specific genes overexpressed in cyclin D3−/− muscle.

Gene symbol	Description	Fold change	q-value
**Sarcomere**			
*Myh7*	myosin, heavy polypeptide 7, cardiac muscle, beta	123,585	2,30E-03
*Myl2*	myosin, light polypeptide 2, regulatory, cardiac, slow	22,073	2,30E-03
*Tnnc1*	slow isoform of Troponin C	21,359	7,50E-02
*Myl3*	myosin, light polypeptide 3	17,760	2,30E-03
*Tnnt1*	troponin T1, skeletal, slow	16,414	2,30E-03
*Tnni1*	troponin I, skeletal, slow 1	13,378	2,30E-03
*Myoz2*	myozenin 2 (calsarcin 1)	3,693	2,30E-03
*Smtnl1*	smoothelin like 1	1,948	2,30E-03
*Actn2*	actinin alpha 2	1,878	2,30E-03
*Xirp1*	xin actin-binding repeat containing 1	1,836	2,30E-03
*Myh3*	myosin, heavy chain 3, skeletal muscle, embryonic	1,576	2,30E-03
**Calcium signaling**			
*Atp2a2*	ATPase, Ca^++^ transporting, cardiac muscle, slow twitch 2	4,574	2,30E-03
*Casq2*	calsequestrin 2 (slow & cardiac)	2,004	2,30E-03
**Nucleus**			
*Fhl2*	four and a half LIM domains 2	5,243	2,30E-03
*Ankrd2*	ankyrin repeat domain 2 (stretch responsive muscle)	2,678	2,30E-03
*Csrp3*	cysteine and glycine-rich protein 3	2,356	2,30E-03
*Ppara*	peroxisome proliferator-activated receptor alpha	2,211	4,26E-03
*Pdlim1*	PDZ and LIM domain 1 (elfin)	1,879	2,30E-03
*Fhl1*	four and a half LIM domains 1	1,800	7,55E-03
*Ppargc1a*	peroxisome proliferator-activated receptor gamma coactivator 1a	1,450	9,36E-03
**Other**			
*Hspa1a*	heat shock protein 1 A	2,685	2,30E-03
*C1qb*	complement component 1, q subcomponent, beta polypeptide	2,434	2,30E-03
*Lmod2*	leiomodin 2 (cardiac)	2,308	2,30E-03
*Sorbs1*	sorbin and SH3 domain containing 1	1,911	2,30E-03
*Khdrbs3*	KH domain containing, RNA binding	1,856	2,30E-03
*Prkd1*	protein kinase D1	1,752	1,81E-02
*Slit3*	slit homolog 3 (Drosophila)	1,749	4,26E-03
*Shisa5*	shisa family member 5	1,560	4,26E-03
*Lrrn1*	leucine rich repeat protein 1, neuronal	1,543	7,55E-03
*Dgat2*	diacylglycerol O-acyltransferase 2	1,520	2,30E-03
*Pcp4l1*	Purkinje cell protein 4-like 1	1,455	2,30E-03
*Mgst1*	microsomal glutathione S-transferase 1	1,367	8,94E-03
*Pla2g12a*	phospholipase A2, group XIIA	1,333	1,81E-02

List of genes upregulated in D3−/− muscle, as identified in RNA-seq, overlapping those specifically expressed in slow myofibers, as determined by Chemello *et al*., 2011.

**Figure 6:**
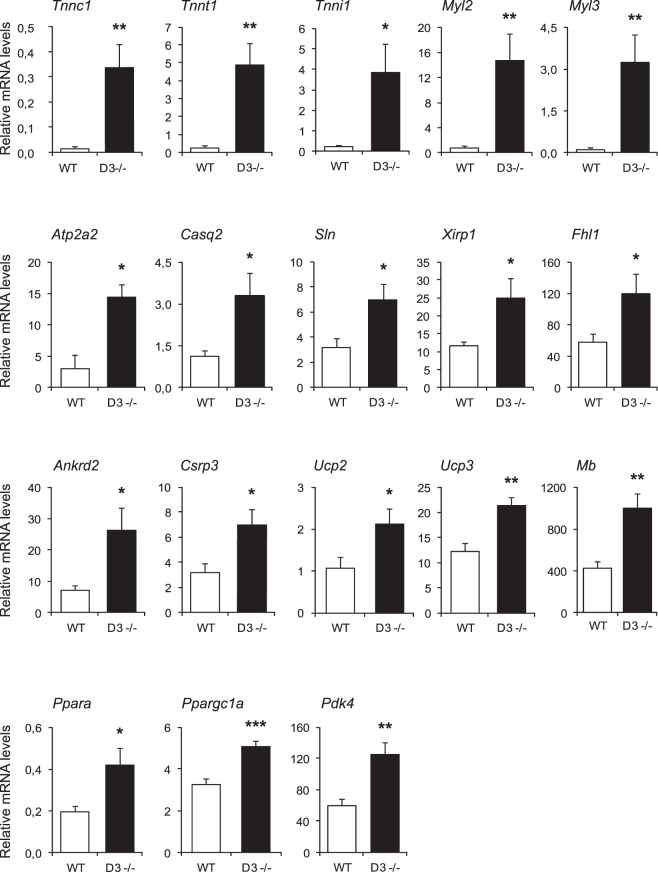
Validation by RT-qPCR of representative cyclin D3 target genes. Expression analysis by RT-qPCR of selected genes in quadriceps muscles isolated from adult D3−/− and WT mice (n = 7). mRNA levels are expressed relative to TBP (TATA binding protein) mRNA, used as endogenous control. Data are reported as means ± SEM. Statistical differences between WT and D3−/− mice was assessed by two-tailed unpaired t-tests (**p < 0.01, ***p < 0.001).

We confirmed by real-time PCR the induction of key biomarkers of oxidative myofibers and oxidative metabolism in quadriceps muscles of D3−/− mice relative to WT mice (Fig. 6). Specifically, we validated the upregulation of transcripts encoding: (*i)* the slow isoforms of troponins (*Tnni1*, *Tnnc1*, and *Tnnt1*) and myosin light chains (*Myl2* and *Myl3*); (*ii)* the slow fiber-specific Ca^2+^ channel SERCA2, the Ca^2+^ binding protein calsequestrin2 and sarcolipin, a regulator of SERCA activity (*Atp2a2*, *Casq2* and *Sln*); (*iii)* sarcomere-associated transcription co-regulators, such as ankyrin repeat domain-containing protein 2 (*Ankrd2*), xin actin-binding repeat containing protein 1 (*Xirp1*), four-and-a-half-LIM domains protein 1 (*Fhl1*) and cysteine and glycine-rich protein 3 (*Csrp3*); (*iv)* the PPARα and PGC1α transcription regulators involved in fatty acid metabolism (*Ppara* and *Ppargc1a*); (*v)* markers of oxidative metabolism, such as the uncoupling proteins 2 and 3 (*Ucp2* and *Ucp3*), the oxygen transport protein myoglobin (*Mb*) and pyruvate dehydrogenase kinase isozyme 4 (*Pdk4*). Although terminal muscle fiber specialization cannot be achieved *in vitro*, the expression of the majority of the above genes was significantly increased also in cyclin D3−/− myogenic precursor cells induced to differentiate *in vitro* as compared to WT myotubes, thus supporting the idea that the effects of cyclin D3 ablation observed in adult muscle are cell autonomous (Supplementary Figure S2).

Collectively, the above data clearly show that cyclin D3 participates in the regulation of basal muscle fiber-type specific transcription programs. To identify upstream regulatory factors whose function might be targeted by cyclin D3, we investigated transcription factor (TF)-binding motifs in the region encompassing 4 Kb around the transcription initiation site of selected genes induced in D3−/− muscle. To this end, the list of genes upregulated in D3−/− muscle selected through GO analysis (Supplementary Table S2), merged to the list of genes enriched in D3−/− muscle and in slow myofibers (Table 2), was subjected to enrichment analysis performed on gene sets derived from the TFT (transcription factor targets) category of the Molecular Signature Database (http://www.broadinstitute.org/gsea/msigdb/index.jsp).

**Figure 7:**
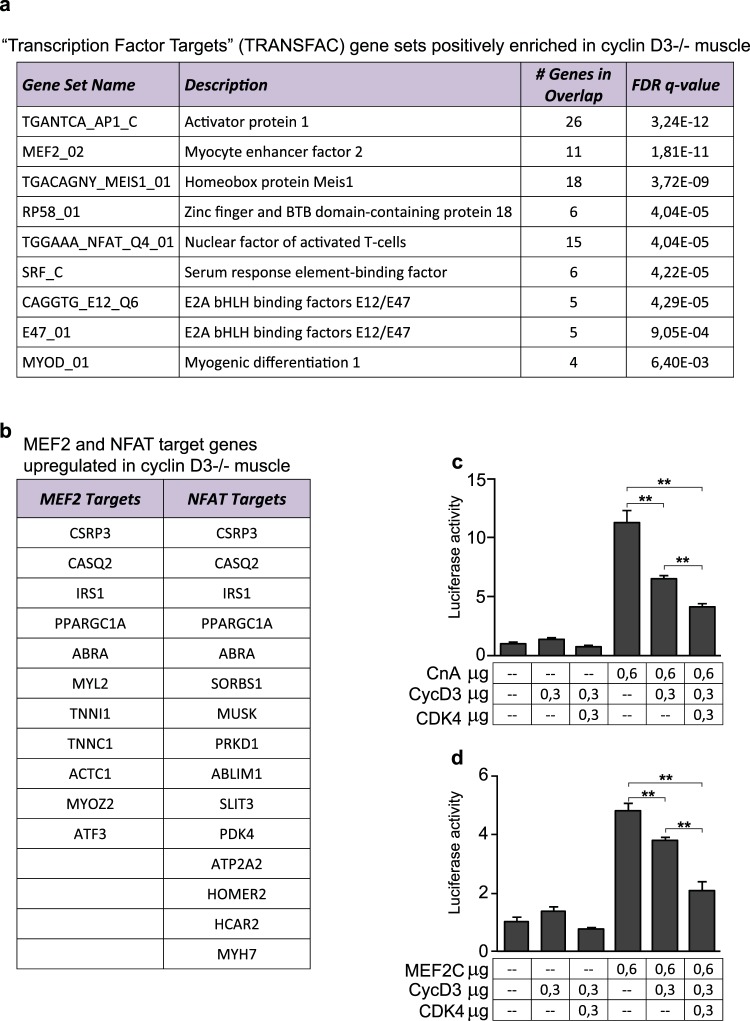
Cyclin D3 regulates the expression of MEF2 and NFAT target genes. (**a**) Transcription factor-binding motifs enriched in the promoter region of genes upregulated in cyclin D3−/− muscle. Enrichment analysis of 77 selected genes upregulated in cyclin D3−/− muscles performed on gene sets derived from the Transcription Factor Targets (TFT) sub-collection of the Molecular Signature Database (MSigDB: http://software.broadinstitute.org/gsea/msigdb/index.jsp). Each TFT gene set is associated to number of genes and FDR q-value (<0.05). (**b**) MEF2 and/or NFAT target genes upregulated in cyclin D3−/− muscle. List of genes upregulated in cyclin D3−/− muscle overlapping the MEF2 and/or NFAT gene sets from the MSigDB TFT collection. (**c**,**d**) Cyclin D3 represses the calcineurin-stimulated (panel c) or MEF2C-stimulated (panel d) activity of the myoglobin (Mb) gene promoter. C2 muscle cells were transiently transfected with the Mb-luciferase reporter plus either empty expression vehicles (–) or the indicated combinations of plasmids encoding activated calcineurin (CnA), MEF2C, cyclin D3 (D3) or CDK4. Data are expressed relative to the luciferase activity measured in the control state (co-transfection with empty vectors), and represent mean values ± SEM from four independent transfections. Statistical differences between WT and D3−/− mice was assessed by two-tailed unpaired t-tests (**p < 0.01).

As shown in Fig. 7 (panel a), a top-ranking gene set upregulated in D3−/− muscle was comprised of genes regulated by the MEF2 family of transcription factors, key regulators of skeletal muscle differentiation that are also implicated in the establishment of slow, oxidative myofiber identity during development and in response to calcium-dependent signaling^53,54^. A second highly enriched gene set included genes regulated by NFAT (nuclear factor of activated T cells), a transcription factor that cooperates with MEF2 to activate slow fiber gene expression in response to calcium-regulated signals^55,56^. Other overrepresented binding sites were those for Pbx/Meis and RP58, which are known to play fundamental roles in the control of skeletal muscle gene expression^57,58^, and for pleiotropic transcription factors such as AP1 and SRF. Therefore, combining the results obtained from TF-binding motif investigation and differential gene expression analysis we could identify a considerable number of MEF2 and/or NFAT target genes which were significantly upregulated in D3−/− muscle as compared to WT muscle (Fig. 7, panel b). These results suggest that cyclin D3 may regulate the fiber type phenotype by acting as a repressor antagonizing the activity of MEF2 and/or NFAT transcription factors.

To address this issue, we performed transient transfection assays in cultured muscle cells using a luciferase reporter gene linked to a well-characterized promoter region from the myoglobin gene (Mb) that includes MEF2 and NFAT binding sites, both of which direct the selective expression of myoglobin in slow and fast/oxidative fibers and are necessary for activation of the Mb promoter in response to calcineurin, a calcium/calmodulin-dependent serine/threonine phosphatase^53,55^. C2 myoblasts were co-transfected with the Mb-luciferase reporter and a plasmid construct encoding a constitutively active (calcium-insensitive) form of calcineurin along with constructs encoding cyclin D3 and CDK4 (Fig. 7, panel c). Cyclin D3 was able to repress the calcineurin-dependent activation of the Mb promoter and the combined effect of cyclin D3 and CDK4 was greater than that produced by cyclin D3 alone. Similar results were obtained following co-transfection of the Mb-luciferase reporter along with an expression construct for MEF2C, with or without the addition of cyclin D3 and CDK4 (Fig. 7, panel d). We conclude that cyclin D3 can repress the calcineurin- or MEF2-stimulated transcription from the Mb promoter in cultured C2 muscle cells with the involvement of the associated CDK4 kinase activity.

## Discussion

Our results disclose a previously unknown function for cyclin D3 in shaping skeletal muscle fiber-types, motor performance and whole-body energy metabolism.

We show that mice lacking cyclin D3 display an increased number of muscle fibers with higher oxidative capacity in fast-twitch muscles typically composed of myofibers that primarily utilize glycolytic metabolism. The higher number of oxidative muscle fibers may contribute to the enhanced fatigue-resistance exhibited by juvenile and adult cyclin D3−/− mice during an endurance-type exercise. These changes could be linked to the reprogramming of global muscle gene expression toward a slower, more oxidative transcription program. In fact, the gene-profiling analysis of quadriceps muscle from adult cyclin D3−/− mice revealed the upregulation of several genes previously identified through microgenomic analysis as specifically enriched in slow-twitch myofibers^52^. These findings strongly suggest that cyclin D3 acts as a negative regulator of factors that promote the slow, oxidative fiber-specific gene program. The observed phenotype of cyclin D3−/− adult muscle is potentially due to the lack of cyclin D3 in developing myocytes. Indeed, cyclin D3 protein was shown to be expressed during the late fetal and early post-natal phases of muscle growth but was undetectable in adult myofibers in homeostatic conditions^40^. Elucidating whether cyclin D3 is reactivated in adult muscle during physiologic or pathologic processes to participate in the adaptive response of muscle to external signals is a challenge for future studies.

The transcripts most upregulated in cyclin D3−/− muscle, and whose induction was validated by RT-qPCR, encode structural and regulatory components of the sarcomere that contribute to the contractile properties of slow and fast-oxidative myofibers. Specifically: (*i)* the slow isoforms of myosin heavy and light chains that together form the thick filament myosin motor; *(ii)* the slow isoforms of troponins, tropomyosin and actin that form the thin filament; (*iii)* the sarcomere-associated transcription co-regulators *Ankrd2*, *Xirp1*, *Csrp3* and *Fhl1*. Among the genes upregulated in cyclin D3−/− muscle we could also confirm the induction of metabolic regulators typically enriched in slow and fast-oxidative myofibers, including the transcription factor PPARα, which plays a major role in promoting fatty acid oxidation and lipoprotein metabolism^59^ and the transcription coactivator PGC1α, a master regulator of mitochondrial oxidative metabolism and muscle fiber type determination^60,61^. Other identified and validated markers of enhanced oxidative metabolism were, the oxygen-carrying protein myoglobin, the mitochondrial uncoupling proteins 2 and 3 (UCP2, UCP3), both of which have been shown to improve fatty acid oxidation and decrease production of reactive oxygen species^62,63^, and PDK4 (pyruvate dehydrogenase kinase isozyme 4), which limits glycolysis-derived pyruvate utilization thus promoting the use of fatty acids as substrate for mitochondrial oxidation. These effects of cyclin D3 on the adult muscle gene profile appear to be cell autonomous, because the induction of the majority of the above genes was maintained in primary cyclin D3−/− myogenic precursors induced to differentiate *in vitro*.

Skeletal muscle is the main organ responsible for the way in which changes in substrate availability affect carbohydrate and lipid metabolism, and hence whole body energy metabolism. Consequently, any change in fiber type composition has a great impact on fuel consumption. Cyclin D3−/− mice exhibit a fast-to-slow shifting in muscle fiber type, and such a remodeling of the myofiber phenotype is accompanied by a hypermetabolic phenotype. In fact, mice lacking cyclin D3 exhibited higher rates of basal and resting energy expenditure, which is indicative of higher rates of oxygen consumption. Moreover, juvenile and adult cyclin D3−/− mice showed a decreased respiratory exchange ratio (RER) in comparison to WT mice, indicating a preferential utilization of lipid over carbohydrate as an energy substrate. It was only under conditions of caloric load due to the exposure to HFD that the energy expenditure of WT animals became equivalent to that of cyclin D3−/− animals in standard feeding conditions. The exposure to HFD further increased the EE in juvenile/adult cyclin D3−/− animals, and emphasized the use of fatty acids as fuel source. Our observations are in line with the previously described cyclin D3−/− mice resistance to HFD-induced obesity^27^.

Oxygen uptake is of key importance for endurance performance and is proportional to muscle fiber oxidative and vascular capacity^64,65^. Juvenile and adult cyclin D3−/− animals performed better than WT in a one bout endurance-type exercise test, but such a higher capacity to perform work under oxidative conditions was lost with age. In basal conditions, aged D3−/− animals still show increased energy expenditure compared with aged WT animals. However, they lose the higher capacity to oxidize fat-based fuels displayed at younger ages, and this may correlate with reduced endurance performance. In fact, increased oxidation of fatty acids reduces carbohydrate utilization, thus sparing glycogen stores, suppresses lactate production and, ultimately, leads to increased endurance performance. Interestingly, recent single muscle fiber proteomic analyses revealed that the mitochondria of different fiber types have diverging capabilities for substrate utilization and that aging affects different muscle fiber types unequally^66,67^. In fact, muscle aging is associated with selective atrophy of fast fibers and diverging metabolic and protein quality control adaptations in slow and fast fibers^67^. In particular, enzymes of carbohydrate metabolism increase in slow and decrease in fast aging fibers. Such age-related remodeling of muscle fiber types might explain, at least in part, the fading of the metabolic and physiological differences observed at younger ages between WT and D3−/− mice.

It should be emphasized that the intrinsic endurance exercise capacity of an organism relies largely on muscle oxidative capacity, but is also influenced by several additional variables, such as for example cardiac and pulmonary function, peripheral vasculature and neuromuscular function. Therefore, we do not rule out the possible contribution of tissues other than skeletal muscle to the improvement in exercise performance observed in D3−/− mice, and/or to its fading with age. The assessment of the specific contribution of skeletal muscle to the physiological properties of D3−/− mice described here (i.e. increased energy expenditure and fatty acid oxidation and improved endurance exercise performance) awaits the generation and analysis of tissue-specific knockout mice.

Importantly, in cyclin D3−/− muscle some transcripts were also upregulated which encode components of the sarcoplasmic reticulum (SR) calcium handling machinery predominantly expressed in slow twitch/fast oxidative myofibers, such as the Ca^2+^ -binding protein calsequestrin 2, the SR Ca^2+^ transport ATPase (SERCA 2/ATP2a2), and sarcolipin (SLN), a regulator of SERCA ATP-ases. Besides its well-defined function in initiating muscle relaxation by Ca^2+^ refilling into the SR, the SERCA pump is involved in contraction-independent energy expenditure or muscle-based nonshivering thermogenesis (NST). SLN is a regulator of SERCA activity that has been recently described as a key determinant of the basal metabolic rate and muscle-based NST^68,69^. SLN inhibits SERCA activity and uncouples Ca^2+^ transport from ATP hydrolysis, increasing ATP hydrolysis and heat production, with a consequent enhancement of mitochondrial oxidative metabolism to support ATP production and heat generation^69^. Furthermore, the SLN uncoupling of SERCA leads to an elevation in cytosolic calcium that serves as a signal to activate downstream signaling pathways, including calcium/calmodulin-dependent kinases and the calcineurin Ser/Thr phosphatase, which play important roles in programming slow/oxidative muscle gene expression^70^.

As for the mechanism(s) through which cyclin D3 might regulate slow and fast-oxidative myofiber gene expression, we show that a considerable number of genes upregulated in cyclin D3−/− muscle, and involved in muscle contraction and metabolism, are targets of MEF2, a family of transcription factors that play a central role as regulators of muscle development by interacting with members of the MyoD family of myogenic regulatory factors to cooperatively activate muscle-specific genes^71^. Among the MEF2 factors, MEF2A is required for early myogenic differentiation, whereas MEF2C has been shown to play an essential role in the perinatal regulation of genes associated with muscle contraction and stress response necessary for proper sarcomere assembly and maintenance of myofiber integrity^72,73^. In addition to its role in muscle development, MEF2 has been shown to serve as a target for the calcineurin phosphatase and calcium/calmodulin-dependent protein kinase to drive oxidative and slow-fiber-specific genes^53–55,70^. Furthermore, calcineurin signaling activates PGC1α, which interacts with and co-activates MEF2^61^. Notably, PGC1α can bind to MEF2 on its own promoter and thus regulate its own transcription through an autoregulatory positive loop^74^.

In addition to MEF2, several genes upregulated in D3−/− muscle are regulated by the NFAT transcription factor, a well-established effector of calcineurin signaling. Indeed, upon calcineurin-mediated de-phosphorylation, NFAT moves from the cytoplasm to the nucleus where it synergizes with other transcription factors, including MEF2, to induce a slow gene program^56,70^.

The above observations suggested that cyclin D3 might act as a negative regulator of the MEF2 and/or NFAT transcriptional activity.

Indeed, as assessed by cotransfection assays in cultured muscle cells, cyclin D3, in cooperation with CDK4, could counteract the calcineurin-dependent activation of the slow fiber-specific myoglobin gene promoter, which is known to be transduced by MEF2 and NFAT transcription factors^53,55^. Moreover, cyclinD3, alone or in combination withCDK4, could also inhibit the activation of the myoglobin promoter mediated by a cotransfected MEF2C expression construct.

Although the underlying molecular mechanism remain to be elucidated, our current data suggest that cyclin D3 inhibits, directly or indirectly, the MEF2 and/or NFAT transcriptional function with the involvement of the associated kinase activity. This hypothesis is also supported by previous evidence showing that cyclin D-CDK4 activity can inhibit the transactivation function of MEF2 factors by blocking their association with the coactivator GRIP-1^75^. Furthermore, a recent unbiased systematic substrate screen identified a broad spectrum of substrates specifically phosphorylated by cyclin D3-CDK6 complexes complexes, including MEF2D, NFATC3 and PGC1α^5^.

Altogether, the data presented here reveal an important role for cyclin D3 in the coordinated regulation of several features of muscle fiber type phenotype as well as overall metabolism and suggest that this function of cyclin D3 depends, at least in part, on its ability to modulate the activity of MEF2 and/or NFAT transcription factors.

## Methods

### Mice

Cyclin D3 knockout mice (D3−/−) were provided by Piotr Sicinski (Dana Farber Cancer Institute, Boston MA). Wild-type (WT) controls were generated along with D3−/− mice by interbreeding heterozygous animals. Mice were genotyped as previously described^32^. Food and water were available ad libitum. Animals were maintained under a 12:12 light/dark cycle, with light on at 07:00 AM. Room temperature ranged from 21 to 24 °C and humidity was kept constant (55 ± 10%). Male mice were used throughout the study.

The experimental protocol was approved by the Ethical Commitee of the IRCCS Santa Lucia Foundation and by the Italian Ministry of Health. All the experiments were conducted in accordance with the Italian National Law (DL 26/2014), with the European Union Council Directive of 22 September, 2010 (2010/63/EU) and regulations on the use of animals for research, and NIH guidelines on animal care.

### Energy metabolism

Energy expenditure (EE), oxygen consumption (*V*O2), and the respiratory exchange ratio (RER) were measured by an indirect calorimeter system (TSE PhenoMaster/LabMaster System^®^) in animals at three different ages: 2–3 (juvenile), 8–10 (adult), and 22–24 (aged) months, by a constant air flow of 0.35 L min. Mice were adapted for 6 h to the metabolic chamber before the start of recording, and *V*O2 was measured every 30 min in each mouse, starting at 7:00 PM and ending automatically after 4 days (96 h later). Room temperature was kept constant (22° ± 1 °C). As index of substrate use, we calculated the ratio between the volume of CO2 produced and the volume of O2 consumed (RER). EE was calculated as EE = (3.815 + 1.232 × *V*C O2/*V* O2) × *V*O2, as provided by the TSE system. The EE and RER for each of the sample points were evaluated across the 96 h of total recording. Locomotor activity was assessed during the indirect calorimetric assay by the number of infrared beams broken. Each cage of the calorimeter system is equipped with the InfraMot^®^ device that uses “passive infrared sensors” to detect and record the motor activity of the mouse by the body-heat image and its spatial displacement across time. Any type of body movement was detected and recorded as activity counts. EE was here also analyzed by considering animals’ steady conditions or lack of motor activity (only values included between 0 and 3 activity counts were included).

### Treadmill running

Exercise studies were performed on a five-lane motorized treadmill equipped with an electronic control unit (Treadmill Model LE8710, PanLab, Comella (BCN), Spain), and an electric shock grid at one end of the treadmill. Shock intensity was set at 0.2 mA. Inclination of the treadmill was set at 10°. The day before exercise testing, the mice were acclimatized to the treadmill by running for 10 min at 10 m/min followed by 2 min at 20 m/min. The exercise testing consisted of a single running session on the treadmill at 10 m/min for 30 min; then the speed was increased by 2 m/min every 15 min. Exercise continued until exhaustion, defined as inability to maintain running speed despite repeated contact with the electric grid. The time for removal of mice from the treadmill was 5 s on the shocker plate without attempting to reengage the treadmill. The time to exhaustion was automatically recorded from the beginning of the running session.

### Cryosections, Histology, and Immunostaining

Freshly dissected Extensor Digitorum Longus (EDL), Tibialis Anterior (TA) and Soleus muscles were embedded in OCT (Optimum Cutting Temperature liquid – Tissue Tek), snap frozen in liquid nitrogen-cooled isopentane, sectioned with 9-μm thickness, and mounted onto poly-L-lysine coated glass slides. NADH dehydrogenase activity was determined by incubation for 30–40 min with 0.4 mg/ml NADH disodium salt and 0.8 mg/ml 4-Nitro Blue Tetrazolium Chloride (Roche Diagnostic) in 0.1 M Tris-HCl (pH 7.5). Immunostaining was performed on serial 9-μm thick, unfixed transverse muscle sections. Sections were permeabilized with 0.4% triton X-100 in PBS for 10 min and incubated in block solution (3% goat serum in PBS) for 60 min, at room temperature. Primary antibodies were diluted in block solution and applied over-night at 4 °C. After two washes in PBS (10 min each), sections were incubated with secondary antibodies, diluted in block solution, for 60 min at room temperature, and then washed twice in PBS. The slides were post-fixed in PFA (4% in PBS) for 10 min, washed with PBS and mounted with glycerol diluted in PBS (3:1). For MHC staining, the following monoclonal antibodies were used as tissue-culture supernatants: BA-D5 (MHC-I; 1:5), SC-71 (MHC-IIa; 1:5), BF-F3 (MHC-IIb; 1:10), 6H1(MHC-IIx; 1:10). The antibodies were from DSHB (Developmental Studies Hybridoma Bank, at the University of Iowa). Anti-laminin rabbit polyclonal antibody (L9393) was purchased from Sigma. Goat anti-Rabbit Alexa-fluor 488, Goat anti-Mouse Alexa-fluor 594, Goat anti-Mouse IgMAlexa-fluor 555 secondary antibodies were purchased from Invitrogen. Goat anti-Mouse IgG1 Biotin-SP-conjugate and Streptavidin Alexa-fluor 488-conjugate were from Jackson ImmunoResearch. Slides were visualized with an epifluorescence Olympus BX53 microscope equipped with a RLT3 camera. For fiber-type analysis, overlapping individual images were assembled with the Image Analysis Software IAS (Delta Sistemi) to reconstruct the entire cross-section, and all fibers were characterized. Cross sectional area (CSA) measurements for MHC-I and MHC-2A fiber types in Soleus muscle were performed by manually outlining about the 80% of total fibers within a muscle cross-section. Fiber type percentages and CSA values are reported as means ± SEM; at least four individuals for each genotype were analysed and a minimum of three sections were counted per biological replica.

### Primary myoblast preparation

Primary muscle cells were derived from hindlimb muscles of two-three month-old mice (two mice for each preparation) as described previously^41^. Briefly, muscles were digested with 0.2% collagenase type-II (Sigma) in DMEM for 30 minutes at 37 °C, and then with 2 mg/ml Collagenase/Dispase (Roche Diagnostic) for 30 minutes at 37 °C. Satellite cells were mechanically dissociated by passing the tissue suspension through a 5 ml pipette; the slurry was sequentially filtered through 70 and 40 μm cell strainers (BD Biosciences) and centrifuged. Pelleted cells were resuspended in F-10 (Gibco) supplemented with 20% FBS, 2.5 ng/ml basis-FGF (Peprotech), and penicillin-streptomycin. The cell suspension was preplated for one hour on uncoated dishes to remove fibroblasts. The medium containing the enriched myoblast population was then plated on tissue culture dishes coated with type-I collagen (Sigma). After three days in culture, primary myoblasts were shifted to differentiation medium and harvested for RNA preparation after 72 hours.

### Plasmid constructs

The Luciferase reporter construct containing 2Kb of the upstream promoter region from the myoglobin gene, and the pSRα-CnA construct encoding a constitutively active form of calcineurin A^76^ were kindly provided by Marina Bouchè (University Sapienza of Rome, Italy). The pCDNA1a-MEF2C expression construct was obtained from Eric Olson (University of Texas Southwestern Medical Center). The pBABE puro-cyclin D3 expression construct was generated and previously described by us^39^. The pRc/CMV-CDK4 expression construct was obtained from David M. Livington (Dana Farber Cancer Institute).

### Cell culture, transfections and reporter gene assays

The C2 line of mouse myoblasts (clone C2/7) was originally obtained by M. Buckingham (Institut Pasteur, Paris). Myoblasts were grown in Dulbecco’s modified Eagle’s medium (DMEM) supplemented with 20% fetal bovine serum and antibiotics (100 U of penicillin and 100 μg of streptomycin per ml); differentiation was induced by replacing medium with DMEM supplemented with 5% horse serum. For transient transfection assays, 8 × 10^4^ cells were plated 24 hours before transfection on 35 mm tissue culture dishes, and transfected with the Mb-luciferase reporter (0,2 μg) and with the indicated expression constructs for a total of 1.5 μg of plasmid DNA using the Lipofectamine Reagent (Invitrogen). The pRL-TK control reporter (Renilla luciferase driven by the timidine kinase promoter) was included in all transfections. After 24 hours, the transfected cells were shifted to differentiation medium. Cells were harvested 48 hours after transfection and luciferase assays were performed using the Dual-Luciferase reporter assay system (Promega) according to the manufacturer’s instructions. Extract protein concentration was determined by the Bradford assay. For each sample, the firefly luciferase activity was normalized to the renilla luciferase activity to correct for variations in transfection efficiency. The fold change in luciferase activity was calculated by dividing each normalized luciferase activity value by the average value of normalized luciferase activity observed in the control state.

### RNA isolation and real-time quantitative reverse transcription PCR (RT-qPCR)

Total RNA was extracted from primary myoblasts induced to differentiate or from homogenized muscle tissue using the Trizol reagent (Invitrogen) according to the manufacturer’s instructions. For RT-qPCR, RNA was retrotranscribed using random hexamer primers and MMLV reverse transcriptase (Invitrogen) according to the manufacturer’s directions. Gene-specific primers were designed with Beacon Design software. Amplification was carried out in triplicates using the SYBR Green chemistry and a 7900HT Fast Real-Time PCR System (Applied Biosystens). Identity of the amplicons was verified by their dissociation curves. The mRNA expression values were normalized to those of the TATA-binding protein gene (TBP) used as endogenous control. Gene specific primer sets used for RT-qPCR are listed in Supplementary Table 3.

### Transcriptome sequencing

For RNA-sequencing, we used total RNA isolated from quadriceps muscles of WT or D3−/− mice (three mice per experimental group). Purified RNA was delivered to IGA Technology Services (www.igatechnoloy.com) for cDNA library preparation (Illumina TruSeq Stranded mRNA Sample Prep kit), RNA sequencing (Illumina HiSeq2500; 50 bp single-end reads, 6-plex run) and standard bioinformatic analysis. Briefly, the CASAVA 1.8.2 version of the Illumina pipeline was used to process raw data for both format conversion and de-multiplexing. Reads alignment to the mm10 genome assembly was done using TopHat/Bowtie tool and gene transcript levels were determined via Cuffdiff in the form of FPKM values.

Gene ontology analyses were performed using the online bioinformatic resource DAVID v6.7 (National Institute of Allergy and Infectious Diseases, NIH)^77,78^, using a P-value of 0.05.

To identify conserved cis-regulatory motifs in the promoter region of selected Cyclin D3-sensitive genes, we performed an enrichment analysis on gene sets derived from the C3 collection (C3: motif gene sets; TFT: Transcription Factor targets) of the Molecular Signature Database (http://software.broadinstitute.org/gsea/msigdb/index.jsp)^79^. Each TFT gene set consists of genes that share highly conserved upstream transcriptional regulatory motifs extracted from v 7.4 TRANSFAC database^80^.

### Statistical Analyses

All data are reported in dot plots or in histograms as mean ± standard error of the mean (SEM). Two-way repeated measures ANOVA was used to analyze EE and RER with genotype (D3−/− and WT) and diet regimen (STD and HFD) as between-subject factors and time (4 days of 24 time bins each) as within-subjects factor. Data comparison between two groups (genotypes) for REE levels was analyzed by two-tailed unpaired *t*-tests. Two-way ANCOVA was used to analyze running time and distance including body weight as covariate in the experiments of exercise endurance capacity: Two-way ANOVA was used to analyze myofiber size in Soleus muscle. Post hoc tests for multiple comparisons data were performed with Bonferroni’s or Fisher PLSD for significance, whenever appropriate. For RT-qPCR analysis of RNA from muscle tissue and for fiber type analyses, data comparison between the two genotypes (D3−/− and WT) was examined using two tailed unpaired *t*-tests. For RT-qPCR analysis of RNA from primary myotubes, data comparison between the two genotypes (three independent cell isolations from D3−/− or WT muscles) was analyzed by two tailed paired *t*-test. In all statistical tests, significance was set at *p* < 0.05. Data analyses were carried out using *GraphPad* Prism 6.0 software.

## Electronic supplementary material

Supplementary information

Supplementary Dataset 1

## Data Availability

RNA-seq data generated and analyzed in this paper have been deposited in the Sequence Read Archive (SRA) database (SRA accession: SRP131481). Entire information about expression data and gene ontology results presented in the main paper are provided in Additional Information files. The data that support the findings of this study are available from the corresponding author on reasonable request.
