# Supplementary material for: Lack of cyclin D3 induces skeletal muscle fiber-type shifting, increased endurance performance and hypermetabolism
Source: Sci Rep. 2018 Aug 24;8:12792. doi: 10.1038/s41598-018-31090-5 (PMC6109157; doi:10.1038/s41598-018-31090-5)
Supplement: Supplementary file 1 — Supplementary information [file 41598_2018_31090_MOESM1_ESM.pdf]

Supplementary Information

Lack of cyclin D3 induces skeletal muscle fiber-type shifting, increased endurance performance and hypermetabolism.

Silvia Giannattasio, Giacomo Giacovazzo, Agnese Bonato, Carla Caruso, Siro Luvisetto, Roberto Coccurello, and Maurizia Caruso

Supplementary Table 1.

RNA-seq FPKM, Fold Change and Gene Ontology results for RNA isolated from Quadriceps muscles of WT and cyclin D3-/- mice

See separate excel File, Table S1.xlsx

Supplementary Table 2. Representative genes upregulated in cyclin D3-/- muscle compared to control muscle

Supplementary Table 3. Primers used for quantitative Real Time PCR

Supplementary Figure S1. Fiber type and fiber size analysis of Soleus muscles from cyclin D3-/- and WT mice

Supplementary Figure S2. Primary myotubes mRNA expression of relevant genes induced in cyclin D3-/- adult muscle

Supplementary Figure legends

Table S2

Representative genes upregulated in cyclin D3-/- muscle

Gene symbol	*Description*	*Fold change*	*q-value*
**Muscle organ development**			
Tnni1	troponin I, skeletal, slow 1	13,378	2,30E-03
Fhl2	four and a half LIM domains 2	5,243	2,30E-03
F2r	coagulation factor II (thrombin) receptor	2,202	2,30E-03
Rcan1	regulator of calcineurin 1	1,982	2,30E-03
Tnc	tenascin C	1,858	2,30E-03
Xirp1	xin actin-binding repeat containing 1	1,836	2,30E-03
Barx2	BarH-like homeobox 2	1,780	1,81E-02
Musk	muscle, skeletal, receptor tyrosine kinase	1,775	2,30E-03
Eng	endoglin	1,711	2,30E-03
Tagln2	transgelin2	1,652	2,30E-03
Eln	elastin	1,571	4,26E-03
**Regulation of muscle contraction**			
Atp2a2	ATPase, Ca^{++} transporting, cardiac muscle, slow twitch 2	4,574	2,30E-03
Sln	sarcolipin	2,448	2,30E-03
Casq2	calsequestrin 2 (slow & cardiac)	2,004	2,30E-03
Adrb2	adrenergic receptor, beta 2	1,815	2,30E-03
Edn1	endothelin 1	1,796	3,74E-02
**Sarcomeric and actin binding proteins**			
Myh7	myosin, heavy polypeptide 7, cardiac muscle, beta	123,585	2,30E-03
Myl2	myosin, light polypeptide 2, regulatory, cardiac, slow	22,073	2,30E-03
Myl3	myosin, light polypeptide 3	17,760	2,30E-03
Tnnt1	troponin T1, skeletal, slow	16,414	2,30E-03
Tpm3	tropomyosin 3, gamma	4,600	2,30E-03
Myoz2	myozenin 2 (calsarcin 1)	3,693	2,30E-03
Dbn1	drebrin 1	2,970	6,05E-03
Myo1g	myosin IG	2,970	1,93E-02
Myom3	myomesin 3	2,782	2,30E-03
Ankrd2	ankyrin repeat domain 2 (stretch responsive muscle)	2,678	2,30E-03
Csrp3	cysteine and glycine-rich protein 3	2,356	2,30E-03
Map1a	microtubule-associated protein 1 A	2,355	8,94E-03
Lmod2	leiomodin 2 (cardiac)	2,308	2,30E-03
Hspb7	heat shock protein family, member 7	2,256	2,30E-03
Mlph	melanophilin	2,248	1,04E-02
Gas7	growth arrest specific 7	2,007	2,30E-03
Lcp1	lymphocyte cytosolic protein 1	1,961	4,62E-02

Smtnl1	smoothelin-like 1	1,948	2,30E-03
Pdlim1	PDZ and LIM domain 1 (elfin)	1,879	2,30E-03
Actn2	actinin alpha 2	1,878	2,30E-03
Syne3	spectrin repeat containing, nuclear envelope family member 3	1,874	7,55E-03
Abra	actin-binding Rho activating protein	1,857	2,30E-03
Mmp2	matrix metallopeptidase 2	1,735	2,30E-03
Homer2	homer homolog 2 (Drosophila)	1,651	2,30E-03
Actc1	actin, alpha, cardiac muscle 1	1,621	2,30E-03
Ablim1	actin-binding LIM protein 1	1,589	2,30E-03
Myh3	myosin, heavy chain 3, skeletal muscle, embryonic	1,576	2,30E-03
Flnb	filamin, beta	1,535	2,30E-03
Gsn	gelsolin	1,506	2,30E-03
**Glucose and lipid metabolism**			
Atf3	activating transcription factor 3	3,392	2,30E-03
Hcar2	niacin receptor1	3,264	2,37E-02
Irs2	insulin receptor substrate 2	2,223	2,30E-03
Ppara	peroxisome proliferator-activated receptor alpha	2,211	4,26E-03
Abcg1	ATP-binding cassette, sub-family G (WHITE), member1	1,937	1,56E-02
Sorbs1	sorbin and SH3 domain containing 1	1,911	2,30E-03
Ogdhl	oxoglutarate dehydrogenase-like	1,855	2,30E-03
Gck	glucokinase	1,849	2,47E-02
Pfkl	phosphofructokinase liver B-type	1,831	2,02E-02
Plin5	perilipin5	1,821	2,30E-03
Ldhb	lactate dehydrogenase B	1,784	2,30E-03
Irs1	insulin receptor substrate 1	1,604	2,13E-02
Pdk4	pyruvate dehydrogenase kinase, isoenzyme 4	1,485	2,30E-03
Cpt1a	carnitine palmitoyltransferase 1a, liver	1,479	1,04E-02
Lpl	Lipoprotein lipase	1,469	2,30E-03
**Miscellaneous**			
Hspa1a	heat shock protein 1A	2,685	2,30E-03
Hspa1b	heat shock protein 1b	2,606	2,30E-03
Ucp2	uncoupling protein 2	2,092	2,30E-03
Esrrb	estrogen receptor-related receptor beta	1,997	2,30E-03
Esrrg	estrogen receptor-related receptor gamma	1,973	2,30E-03
Fgf1	fibroblast growth factor 1	1,756	8,95E-03
Mb	myoglobin	1,747	2,30E-03
Vegfa	vascular endothelial growth factor A	1,601	1,18E-02
Ppargc1a	peroxisome proliferator-activated receptor gamma coactivator 1a	1,450	9,37E-03
Ucp3	uncoupling protein 3	1,383	1,44E-02

Table S3

Sequences of the primers used for quantitative PCR analysis

Gene	***Accession Number***	***Forward primer***	***Reverse primer***
Myh1	NM_030679	AAAGCTTCAAGTTTGGACC	GAGAGAGCCTGCCTTTAG
Myh4	NM_010855	GTCCTTCCTCAAACCCTTA	CTCTTGTGTTACCAGATGAAG
Myh7	NM_080728	CCTGCTGTTTCCTTACTTG	CTGTACTCCTCTGCTGAG
Myh2	NM_001039545	CTGACTCGTCCTGCTTTA	CTCACAGACCCTTACTGG
Ppargc1a	NM_008904	CGGTCTTAGCACTCAGAA	AATTCTCGGTCTTAACAATGG
Ucp2	NM_011671	AAGCAGCCTCCAGAACTC	GACCTTCAATCGGCAAGAC
Ucp3	NM_009464	TACCCAACCTTGGCTAGACG	GTTCCTTTGCTGCCTATGGA
Pdk4	NM_013743	AGCTGCTGGACTTTGGTTCA	CGGTCAGGCAGGATGTCAAT
Ankrd2	NM_020033	CAGGAGGAAGAGACTGAGAAACT	TAAAGCAGGACTCTGAACCCC
Fhl1	NM_001077361	GAAGTGCAACAAGGCCATCAC	GCCAGCTTCTTAGAGCAGGTAA
Xirp1	NM_011724	GGTTACTCAAGGCAGCATCA	CTTCTGTCACAGCAGCTTTCT
Mb	NM_001164047	AGGAAGTCCTCATCGGTCTGT	GGTCCTCTGAGCCCTTCATA
Ppara	NM_011144	GCATTTGGGCGTATCTCAC	CTTCAACTTGGCTCTCCTCTA
Atp2a2	NM_009722	AACCTTTGCCGCTCATTTTCC	CATGAGGATCACAGGCAAGGA
Casq2	NM_009814	ACATCAAAGACCCACCCTACG	CCCATTCAAGTCGTCTTCCCA
Tnnt1	NM_001277903	ATCTGTGGACCCAGCCTTAG	CTCTTCTCGCTCTGCCACC
Tnnc1	NM_009393	GCCTGTCCTGTGAGCTGTCT	CAGCTCCTTGGTGCTGATG
Tnni1	NM_021467	GCACTTTGAGCCCTCTTCAC	AGCATCAGGCTCTTCAGCAT
Myl2	NM_010861	ACTATGTCCGGGAGATGCTG	TCTCCGTGGGTAATGATGTG
Myl3	NM_010859	AAGAAGGATGATGCCAAAGC	TCGATCTTAATCTTGGAGGCA
Csrp3	NM_013808	GCAGTTCCAACAATCCCCAAA	GGCACTTCTCTGATTCTCCAAAC
Sln	NM_025540.2	CTCCTCTTCAGGAAGTGAAGACA	GCTCCTGAGTAGACCTCTCCAT
Tbp	NM_013684	CCAATGACTCCTATGACCCCTA	CAGCCAAGATTCACGGTAGAT

Supplementary Figure S1

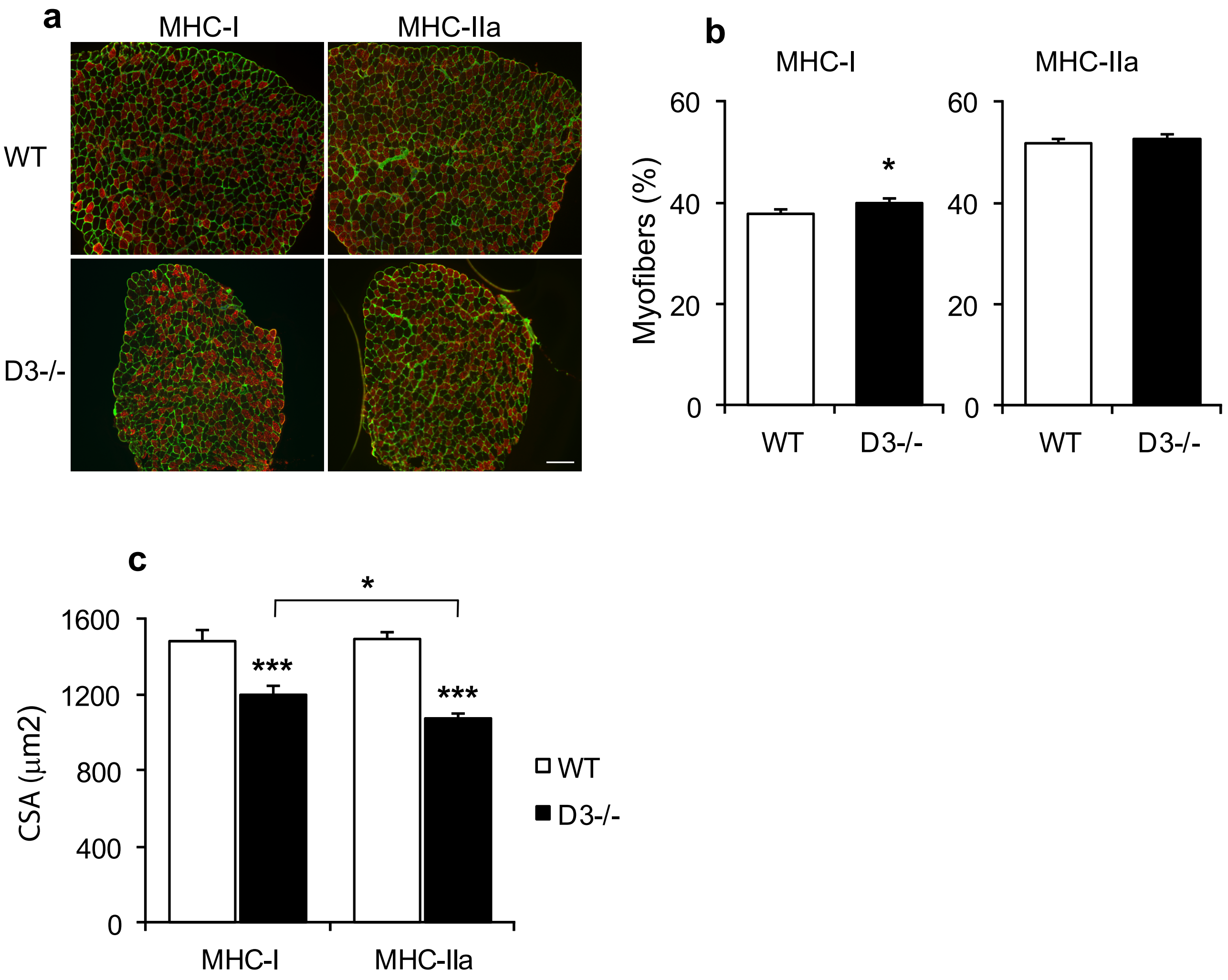

Supplementary Figure S2

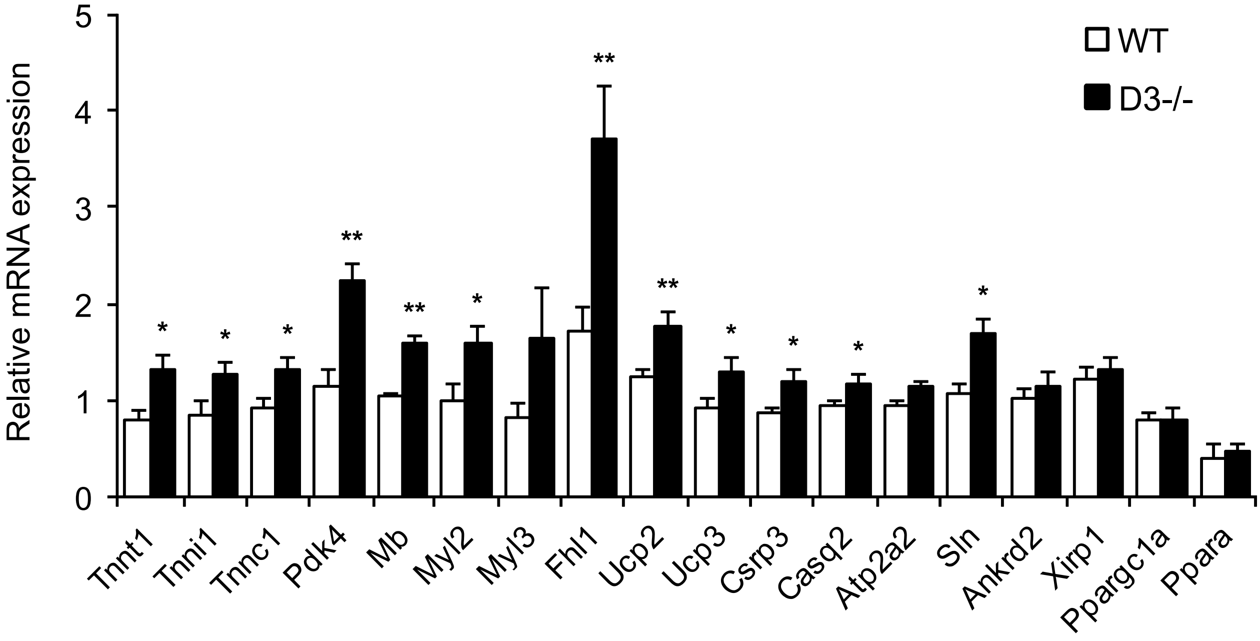

Supplementary Figure S1. Immunoistochemical analysis of MHC isoforms in Soleus muscles from cyclin D3-/- and WT mice.

(**a**) Co-immunostaining for laminin (green) and either MHC-I or MHC-IIa (red) in serial sections of WT and D3-/- Soleus muscle. (**b**) Quantification of MHC-I and MHC-IIa fibers in Soleus serial cross sections (WT, n=5; D3-/-, n=6). The data are expressed as the percentage in each category of the total number of fibers per section and are presented as means ± SEM. Quantification was carried out in least three sections per individual mouse. Statistical differences between WT and D3-/- mice were assessed by two-tailed unpaired t-tests (*$p<0.05$). (**c**) Cross sectional area (CSA) of MHC-I and MHC-IIa fibers in Soleus muscles of WT (n=5) and D3-/- (n=6) mice. Data are presented as means ± SEM. For each genotype, at least 3 sections per individual were analyzed and the data was averaged (total number of myofibers measured: WT, 3977 MHC-I and 4320 MHC-IIa myofibers; D3**-/-,** 2279 MHC-I and 3606 MHC-IIa myofibers). Statistical analysis of average CSA per section was carried out by two-way ANOVA (genotype effect $F[1,71] = 71.685$, $p < 0.0001$), followed by Fisher PLSD *post-hoc* comparison of D3-/- vs WT mice or MHC-I vs MHC-IIb myofibers. Asterisks denote significance by PLSD test (***$p < 0.001$, *$p<0.05$). Scale bar: 100 μm.

Supplementary Figure S2. Primary myotubes mRNA expression of relevant genes induced in cyclin D3-/- adult muscle

Expression analysis by RT-qPCR of selected genes in WT and D3-/- myogenic precursor cells induced to differentiate *in vitro*. mRNA levels are expressed relative to TBP (TATA binding protein) mRNA, used as endogenous control. Data are reported as means ± SEM of three independent experiments (n=3 cell preparations/genotype). Statistical differences between WT and D3-/- primary myotubes was assessed by two-tailed paired t-tests (* $p<0.05$, ** $p<0.01$).
